# Norepinephrine influences the circadian clock in human dermal fibroblasts from study participants with a diagnosis of attention-deficit hyperactivity disorder

**DOI:** 10.1007/s00702-021-02376-2

**Published:** 2021-07-18

**Authors:** Denise Palm, Adriana Uzoni, Frederick Simon, Oliver Tucha, Johannes Thome, Frank Faltraco

**Affiliations:** Department of Psychiatry and Psychotherapy, University Medical Centre Rostock, Rostock, Gehlsheimer Str. 20, 18147 Rostock, Germany

**Keywords:** Norepinephrine, Human dermal fibroblasts, Circadian rhythm, Attention-deficit hyperactivity disorder

## Abstract

Attention-deficit hyperactivity disorder (ADHD) is characterized by changes to the circadian process. Many medications used to treat the condition, influence norepinephrine levels. Several studies have, in addition, reported that norepinephrine itself has an effect on circadian function. The aim of this study was to investigate the circadian gene expression in primary human-derived dermal fibroblast cultures (HDF) after norepinephrine exposure. We analyzed circadian preference, behavioral circadian and sleep parameters as well as the circadian gene expression in a cohort of healthy controls and participants with an ADHD diagnosis. Circadian preference was evaluated with German Morningness–Eveningness Questionnaire (D-MEQ) and rhythms of sleep/wake behavior were assessed via actigraphy. After *ex vivo* exposure to different norepinephrine concentrations in HDF cultures, the rhythmicity of circadian gene expression was analyzed via qRT-PCR. The exposure of 1 µM norepinephrine to confluent cultures of human dermal fibroblasts from participants with a diagnosis of ADHD, was shown to dampen *Per1* rhythmicity. The expression of *Bmal1*, *Per1* and *Per3* in control subjects was also influenced by incubation with 1 µM norepinephrine. Cultures from the ADHD group revealed no statistically significant overall differences in circadian gene expression, between cultures with and without norepinephrine incubation. *Per3* expression showed a significant ZT × group interaction via mixed ANOVA. *Per3* expression at ZT4 was significant higher in the group of control samples incubated with 1 µM norepinephrine, compared to the control group without norepinephrine. This effect was also shown in the control samples incubated with 1 µM norepinephrine and cultures from subjects with ADHD without norepinephrine incubation. *Per3* expression differed between the healthy control group and the ADHD group without norepinephrine incubation at ZT28. The results of the present study illustrate that norepinephrine impacts on circadian function. In both groups, control group and cultures taken from subjects with ADHD, the expression of the *periodic genes* (*Per1–3*) was significantly influenced by incubation with norepinephrine.

**Supplementary Information:**

The online version contains supplementary material available at 10.1007/s00702-021-02376-2.

## Introduction

The regulation of the norepinephrine (NE) homeostasis as well as the NE re-uptake into presynaptic nerve terminals among other substances is mediated by the NE transporter (NET) (Xu et al. 2000). Norepinephrine, like dopamine and epinephrine, is a catecholamine neurotransmitter derived from the common precursor amino acid tyrosine. Tyrosine is converted to l-dihydroxyphenylalanine (Dopa), after which Dopa is then converted to dopamine and transported to synaptic vesicles. Dopamine is converted to epinephrine, which is released into the cytosol for conversion to NE and after pre-synaptic release interacting with adrenoceptors (Xing et al. 2016). NE binds to the α2-adrenoceptors (α2A, α2B and the α2C) with highest affinity. In the skin, NE also binds to β2-adrenoceptor expressed on the surface of keratinocytes, dermal fibroblasts and melanocytes (Gillbro et al. 2004; MacDonald et al. 1997; Steinkraus et al. 1996).

NE has been demonstrated to influence mammalian circadian rhythmicity. The human inner clock is generated by a master central clock in the suprachiasmatic nuclei (SCN) of the hypothalamus. Circadian rhythm is regulated by the main circadian genes (*circadian locomotor output cycle kaput gene—Clock*, *brain and muscle Arnt-like 1 gene—Bmal1*, *periodic genes—Per1/2/3* and *cryptochrome genes—Cry1/2*) that exhibit an auto-regulatory negative feedback loop, processed by signals like cyclic hormone production, as well as influenced by environmental changes such as light exposure (Czeisler et al. 1999; Moore 1997; Reppert and Weaver 2002). NE is partly responsible for the metabolic activity of the pineal gland and the nocturnal melatonin stimulation (Simonneaux and Ribelayga 2003; Terbeck et al. 2016).

Transient expression of *Per1* in astrocytes is induced *in vitro* by β2-adrenoceptor activation through NE (Morioka et al. 2010). Studies have also demonstrated that NE can reactivate the circadian rhythm of adult rat cardiomyocytes, as well as regulate the physiological expression of *mPer1*, *mPer2* and *mBmal1* in mice livers, and the rhythmic oscillation of *gBmal1*, *gClock*, *gCry1* and *gCry2* in chicken pineal glands (Durgan et al. 2005; Li and Cassone 2015; Terazono et al. 2003). Andrade-Silva et al., a group that studied the circadian gene expression in rat pineal gland cultures in respect to NE synchronization, concluded that NE synchronization mimics its natural release in pineal glands (Andrade-Silva et al. 2014).

Several studies based on cell culture models have demonstrated that alterations in circadian gene expression as well as circadian rhythm disturbances are associated with neuropsychiatric disorders (Cronin et al. 2017; Johansson et al. 2016; Lippert et al. 2014; Mansour et al. 2017; McCarthy et al. 2013a, b; Yang et al. 2009). Alterations in *Per2* and *Cry1* expression between individuals with a diagnosis of ADHD with no medication compared to medicated and healthy controls using human dermal fibroblasts as cellular model were reported (Coogan et al. 2019).

Human-derived fibroblasts provide an advantageous model to study the influence of drugs on circadian gene expression. The synchronization of the circadian system of fibroblasts can be synchronized by different substances, e.g. dexamethasone or NE. The effects of synchronizers are different on circadian gene expression (Faltraco et al. 2020).

NE also affects neurodegenerative and other psychiatric disorders (Marien et al. 2004). It is involved in the modulation of attention, arousal and cognition. Disturbances in the NE network are hypothesized to be associated with the pathophysiology of attention-deficit hyperactivity disorder (ADHD) (Sharma and Couture 2014). Abnormal regulation in NE neurotransmission as well as polymorphisms in the *NET* gene is reported to provide a link between NE and ADHD. In this respect, NE has been suggested to be associated with attention, alertness and executive function. In ADHD, inattentiveness and disturbances in executive functions are characteristic symptoms (Beane and Marrocco 2004; Biederman and Spencer 1999).

This leads to anticipation of an association between NE regulation and circadian gene expression as well as ADHD; and we hypothesized, that NE exposure influences the expression of circadian genes and thus may influence sleep quality and ADHD symptoms. Goal of this study is to investigate the effects of NE on circadian rhythmicity using the fibroblast model.

## Materials and methods

### Participant selection criteria

Ethical approval for the conduct of the study, including obtaining human dermal biopsy samples, was given by the ethical review committee of Rostock University (Registration-number: A2013-159) and written consent was obtained from each study participant. The study was conducted according to the ethical guidelines of the Declaration of Helsinki.

Subjects with ADHD and healthy controls participating in the study were recruited via the Department of Psychiatry and Psychotherapy, University Medical Centre Rostock. All subjects with ADHD were diagnosed by experienced psychiatrists in advance. The control group was recruited of acquaintances of people involved in the study.

Human dermal fibroblasts (HDF) were obtained from skin biopsies from dorsal forearm of subjects with ADHD and control volunteers. Only adult individuals, able to give informed consent, were included. Controls without a history of childhood or adult ADHD were matched for sex and age. Patients with debilitating psychiatric symptoms were excluded. Shift workers were also excluded. Screening for ADHD symptoms was done using the WURS-k (Wender Utah Rating Scale) as well as assessment of symptoms according to DSM-IV and ICD-10 criteria. Additionally, the following psychometric tests were used to confirm ADHD diagnosis: SKIDI and II (structured clinical interview), DIVA 2.0 (structured diagnostic interview), CAARS (Conners’ Adult ADHD Rating Scales) and PSQI (Pittsburgh Sleep Quality Index). The IQ of the healthy control group and volunteers with ADHD diagnosis were measured using MWT (Multiple-Choice Word Test). The chronotypes of the participants were determined by the D-MEQ (Morning–Eveningness Questionnaire, German Version). No special cognitive testing was implemented in the study. Comorbidities were observed: 28.6% of participants with ADHD diagnosis has additionally adipositas, 7.1% has additionally addiction disorder, and 21.4% has additionally affective disorder. The remaining participants with ADHD diagnosis had no comorbidities.

The four manuscripts of this special issue dealing with circadian rhythmicity describe unique research questions (Faltraco et al. 2021a, b, c). Although some samples have been used for more than one research question, the overall sample composition differs from each other and thus is different for each study. Experiments differ substantially in their conditions, thus, they each investigate unique cellular biochemical pathways.

### Actigraphy

To obtain objective measures of participants’ sleep and circadian rhythm function, the rest–activity pattern of participants was recorded using wrist-worn actigraphs (Actiwatch 2, Philips Respironics, USA). Actigraphs were worn on the non-dominant wrist for a period of at least 7 consecutive days. The recording interval of the device was set at 60-s epochs. Data occurring before the first and after the final midnight of each record were excluded, ensuring at least 6 complete days for each participant, with a complete weekend included in each record.

### Tissue isolation and fibroblast cell culture

Human dermal fibroblasts (HDF) were isolated and cultured as described previously (Takashima 1998). Fibroblasts were cultivated (37 °C, 5% CO_2_) in Dulbecco’s Modified Eagle Medium DMEM (Gibco, Thermo Fisher, UK)/1 mg/ml Liberase™ (Roche, Germany) containing 100 units/ml penicillin, 100 µg/ml streptomycin (Gibco, Thermo Fisher, UK) and 10% fetal bovine serum FBS (Gibco, Thermo Fisher, UK).

### Measurement of cell viability

Upon confluency of the respective primary fibroblast cell culture from each participant, cells were incubated with 0 µM, 0.1 µM and 1.0 µM norepinephrine (Arterenol, Sanofi-Aventis, Germany). Following 24 h, cell viability was measured using the Trypan Blue Exclusion Test (Strober 2015).

### Measurement of circadian gene expression

Upon confluency of the respective primary fibroblast cell culture from each participant, eight-culture-flask replicates were prepared and cells were incubated with either 0.1 µM or 1.0 µM norepinephrine (NE, Arterenol, Sanofi-Aventis, Germany). Cultures without NE were used as a negative control. After 24 h of incubation, the cells were synchronized with 100 nM dexamethasone (Sigma-Aldrich, Germany) for 30 min. Samples were harvested every fourth hour after synchronization for a period of 28 h in solution D (4.5 M guanidinium thiocyanate, 0.5% sodium-*N*-lauryl sarcosine, 25 mM tri-sodium citrate, 0.1 M betamercaptoethanol) and stored at − 70 °C. Total RNA was isolated and purified with RNeasy Plus Mini Kit (Qiagen, Germany) as well as subjected to reverse transcription using the Superscript III First-Strand Synthesis System (Invitrogen, Germany). Gene expression of *Clock*, *Bmal1*, *Per1*, *Per2*, *Per3* and *Cry1* as well as housekeeping genes (*Rpl13A*, *Rpl19A*, *GAPDH*) was measured by real-time quantitative reverse transcriptase polymerase chain reaction (qRT-PCR) with CFX Connect™ Real-Time PCR Detection System (Biorad, Germany). The oligonucleotide sequences are presented in Table 1. All primers were purchased from Eurofins (Alameda, CA). The qRT-PCR was performed in 96-well 0.1-ml thin-wall PCR plates (Applied Biosystems) in the CFX Connect™ Real-Time PCR Detection System (Biorad, München, Germany). Each 20 µl reaction contained 10 µl Kappa SYBR Green Master Mix (Kappa Biosystems, Darmstadt, Germany), 200 nM gene-specific forward and reverse primer mix (Eurofins, Alameda, CA) and 20 ng template DNA. The expression levels of genes of interest were normalized to the geometrical mean of expression level of housekeeping genes *Rpl13A*, *Rpl19A*, and *GAPDH* from the same sample (Mane et al. 2008). The primer efficiency (between 1.89 and 2.00) was evaluated using the RegLinePCR v 11.0 (Heart Failure Research Center). Data were analyzed using the ΔΔCt method (Livak and Schmittgen 2001). The values were normalized to corresponding individual averages.

**Table 1:** Oligonucleotides for qRT-PCR to measure circadian gene expression

Gene	Forward primer (5′-3′)	Reverse primer (5′-3′)
*Clock*	CCAGCAGTTTCATGAGATGC	GAGGTCATTTCATAGCTGAGC
*Bmal1*	AAGGATGGCTGTTCAGCACATGA	CAAAAATCCATCTGCTGCCCTG
*Per1*	TGGGGACAACAGAACAGAGAA	AGGACACTCCTGCGACCA
*Per2*	GTATCCATTCATGCTGGGCT	TCGTTTGAACTGCGGTGAC
*Per3*	TCAGTGTTTGGTGGAAGGAA	TCTGGGTCAGCAGCTCTACA
*Cry1*	CACGAATCACAAACAGACGG	TACATCCTGGACCCCTGGT
*RPL13a*	GCCAGAAATGTTGATGCCTT	AGATGGCGGAGGTGCAG
*RPL19a*	GTGGCAAGAAGAAGGTCTGG	GCCCATCTTTGATGAGCTTC
*GAPDH*	GAAGGTGAAGGTCGGAGT	GAAGATGGTGATGGGATTTC

### Statistical methods

Circadian gene expression data were tested for significant circadian rhythmicity, using CircWave v. 1.4 software (generated by Dr. Roelof Hut; http://www.euclock.org) to determine the best-fitting linear harmonic regression with an assumed period of 24 h and with *α* set at 0.05. The center of gravity of each best-fitting waveform in CircWave was used as the circadian acrophase, and the associated estimation error was used as the SD. Inferential statistics were carried out in SPSS (IBM Corporation).

Actigraphic data were analyzed via MANCOVAs, with age, sex and in some cases ADHD symptom severity included in the model as covariates.

qRT-PCR clock gene data were analyzed via ANOVA and mixed between–within ANOVAs. For all inferential tests, *p* < 0.05 was used to indicate a statistically significant group-wise difference. Sample sizes were calculated via GPower 3.1 software; for correlations, the assumptions used were significance level of *α* = 0.05 and the power of 0.8 for two groups (ADHD, HC) with three measures (0 µM, 0.1 µM and 1 µM NE). Although research in this field is generally scarce, we assumed that the influence of NE on the circadian gene expression will have an effect size *d*′ = 0.5, returning a required total sample size of 21. Taking into consideration an expected drop-out rate, *n* = 12 participants were allocated to each group. One-way ANOVA was used to assess differences of clock gene expression levels among chronotype groups. Associations between clock gene expression and chronotype obtained from the healthy controls and ADHD participants were studied by Spearman’s rank order correlation. Data were analyzed via time series statistics adequately powered by 12 samples each, which in this statistical model is mathematically sufficient and thus representative (Menet et al. 2012; Thaben and Westermark 2016).

## Results

### Demographic data

Human dermal fibroblasts (HDF) were obtained via skin biopsy from healthy controls (HC) (4 men, 8 women; 41.50 ± 14.04 years, mean ± SD; BMI: 25.87 ± 5.42 kg/m^2^, mean ± SD) and volunteers with diagnosed ADHD (9 men, 5 women; 41.57 ± 13.45 years, mean ± SD; BMI: 26.21 ± 3.62 kg/m^2^, mean ± SD). All participants completed the Multiple-Choice Word Test (IQ score: HC: 110.25 ± 9.32, mean ± SD; ADHD participants: 108.86 ± 12.60, mean ± SD, n.s), Morningness–Eveningness Questionnaire, German Version (D-MEQ score: HC: 58.83 ± 8.97, mean ± SD; ADHD participants: 46.57 ± 15.44, mean ± SD, *p* = 0.024) and Wender Utah Rating Scale, German Short Version (WURS-k score: HC: 7.17 ± 8.19, mean ± SD; ADHD participants: 37.21 ± 15.20, mean ± SD, *p* < 0.001). The demographic data are presented in Table 2.

**Table 2:** Demographic data

Demographic data	Healthy controls, *n* = 12	ADHD, *n* = 14
Age	41.50 ± 14.04 years	41.57 ± 13.45 years
Female	8 (66.7%)	5 (35.4%)
BMI	25.87 ± 5.42	26.21 ± 3.62
IQ score	110.25 ± 9.32	108.86 ± 12.60
D-MEQ	58.83 ± 8.97*	46.57 ± 15.44*
WURS-k-score	7.17 ± 8.19***	37.21 ± 15.20***

*p < 0.05, ***p < 0.001

There were no significant differences in age, BMI, IQ or gender across the two study groups. D-MEQ scores indicated that ADHD patients displayed more definitive and moderate evening preference than healthy controls.

### Actigraphy

Measures from the non-parametric circadian rhythm analysis were analyzed across the two groups, healthy controls and ADHD participants, in a MANCOVA with age and sex as co-variates. For two participants, actigraphy analysis was not completed. No statistical significant effect of group was observed (Pillai’s trace = 0.205; *F* = 0.643; *p* = 0.695; partial ETA squared = 0.205). Bonferroni post hoc correction showed no significant difference for mid-sleep on weekend days (*p* = 0.774), mid-sleep on weekdays (*p* = 0.382), social jetlag (*p* = 0.553), sleep efficiency (*p* = 0.975), WASO (wakening after sleep onset; *p* = 0.927) and total number of wake bouts (*p* = 0.659).

**Fig. 1:**
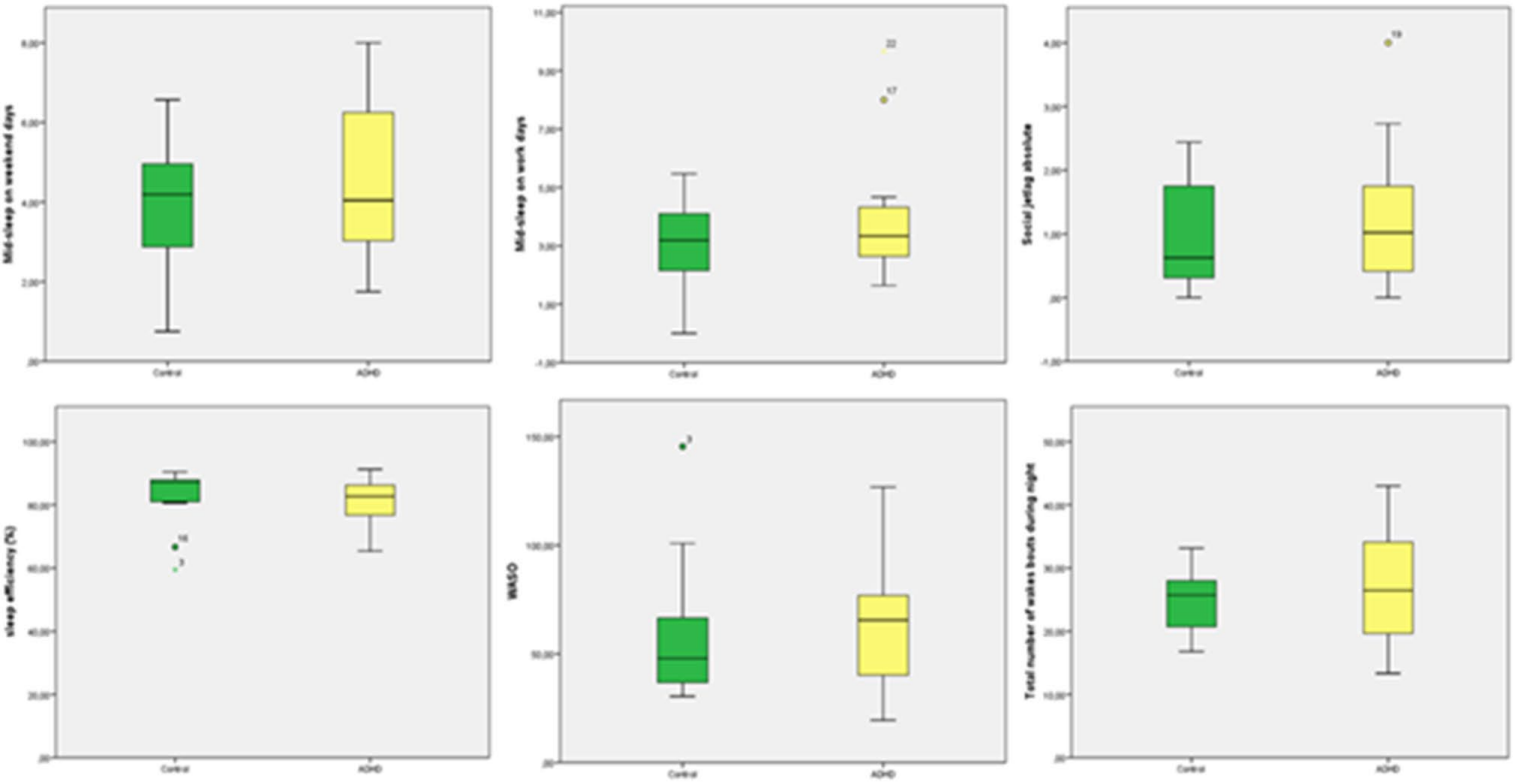
Actigraphic measures of mid-sleep of weekend days, mid-sleep of week days, social jetlag, sleep efficiency, WASO (wakening after sleep onset) and total number of wake bouts are displayed as boxplots. Circles correspond to outlier values and asterisks correspond to extreme values

Measures from the non-parametric circadian rhythm analysis were analyzed across the two groups, healthy controls and ADHD participants, in a MANCOVA with chronotype as co-variate. No statistical significant effect of group was observed (Pillai’s trace = 0.116; *F* = 0.351; *p* = 0.899; partial ETA squared = 0.116). A significant difference for mid-sleep on weekend days (*p* = 0.008), mid-sleep on weekdays (*p* = 0.001), but not for social jetlag (*p* = 0.928), sleep efficiency (*p* = 0.715), WASO (wakening after sleep onset; *p* = 0.925) and total number of wake bouts (*p* = 0.570) was observed (Fig. 1).

### Cell viability

The viability of the cultivated human dermal fibroblasts (HDF) after norepinephrine (NE) incubation was compared with HDFs without NE. The viability of cells treated with NE (0.1 µM NE: 94.01 ± 1.73, mean ± SD; 1.0 µM NE: 93.23 ± 2.19, mean ± SD) was marginally decreased compared to control cells without NE (0 µM NE: 95.44 ± 1.19, mean ± SD).

### Circadian gene expression in human dermal fibroblasts

The expression profiles of six circadian genes after incubation with different NE concentrations were examined in primary fibroblasts cultured from skin biopsies and synchronized with dexamethasone. *Bmal1*, *Cry1*, *Per2*/*Per3* expression was strongly rhythmic in both groups (CircWave, *p* < 0.01). No rhythmicity was detected for *Clock* in both groups except for HC group with 0.1 µM NE (CircWave, *p* = 0.015). In the ADHD group, 1 µM NE exposure dampened the rhythmicity of *Per1* (CircWave, *p* > 0.05).

Gene expression in healthy participants revealed a statistical significant difference between cultures incubated with NE and negative controls (without NE incubation), as determined by one-way ANOVA for *Bmal1* at ZT12 (*F* = 5.043, *p* = 0.012), *Per1* at ZT20 (*F* = 4.730, *p* = 0.016) and *Per3* at ZT4 (*F* = 5.594, *p* = 0.008) and ZT28 (*F* = 3.674, *p* = 0.037). A Bonferroni post hoc analysis revealed a significant lower expression in cultures incubated with 1 µM NE compared to negative controls for *Bmal1* (ZT12, *p* = 0.029), *Per1* (ZT20, *p* = 0.018) and *Per3* (ZT28, *p* = 0.045). The *Bmal1* expression at ZT12 (*p* = 0.029) was significant lower among the cultures incubated with 0.1 µM and 1 µM NE. The *Per3* expression was significant higher at ZT4 (*p* = 0.007) between cultures incubated with 1 µM NE and negative controls. One-way ANOVA in the ADHD group revealed no statistical significant differences between cultures incubated with NE and negative controls (Supplement Fig. S1).

**Fig. 2:**
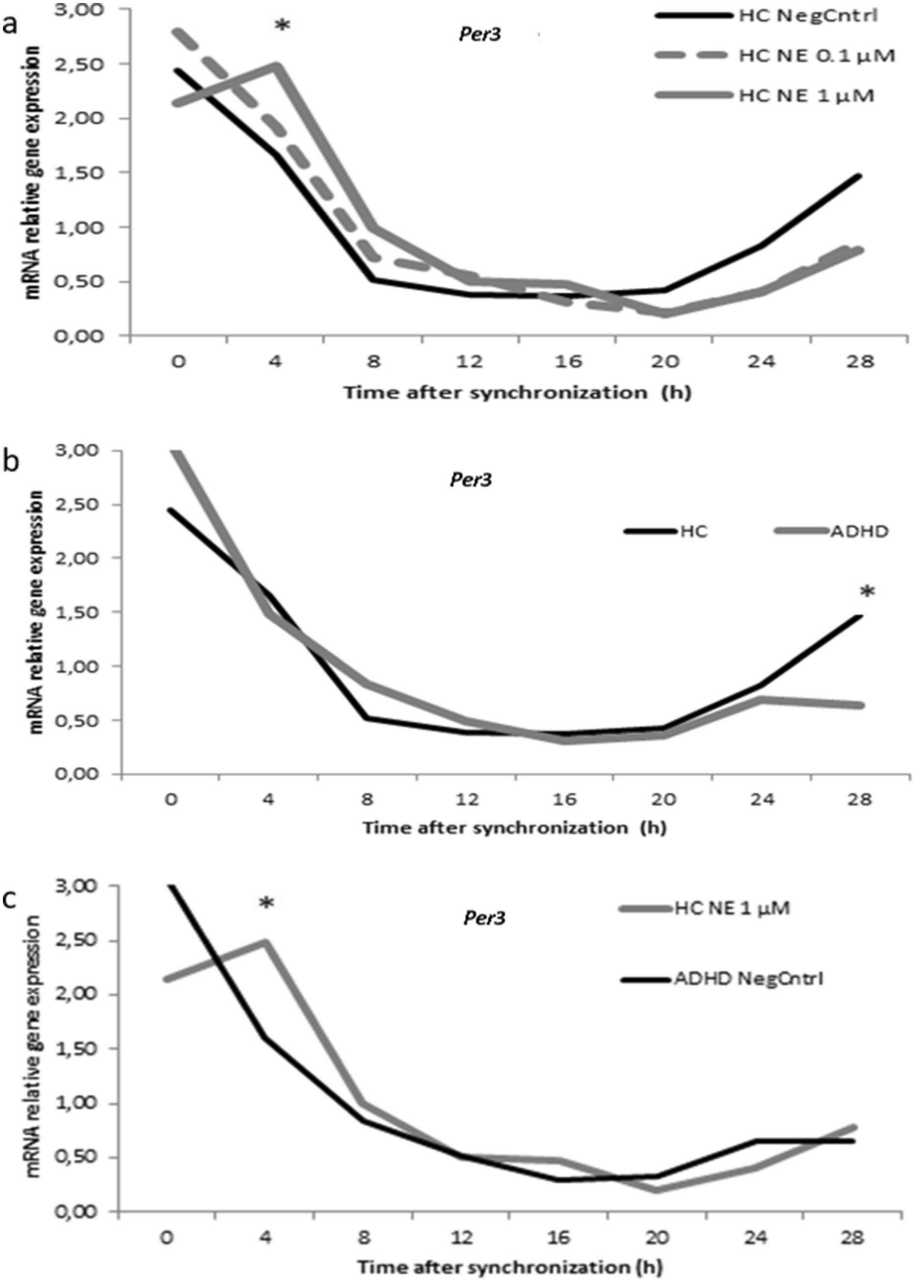
**a** Relative mRNA gene expression of *Per3* in healthy controls (0, 0.1, 1 μM NE). **b** Relative mRNA gene expression of *Per3* between healthy controls and ADHD volunteers for 0 μM NE. **c** Relative mRNA gene expression of *Per3* between healthy controls (1 μM) and ADHD volunteers for (0 μM NE). Asterisks correspond to significant values

Mixed between–within ANOVA analysis of circadian gene data with group as between-subjects factor and time as within-subject factor showed significant main effects for time for all circadian genes (*p* < 0.01). *Per3* expression did show a significant ZT × group interaction via mixed ANOVA (Greenhouse–Geisser corrected *F*_2.58,11.87_ = 1.856, *p* = 0.046, partial ETA squared = 0.140). Bonferroni post hoc correction showed *Per3* expression at ZT4 to be significant higher in the healthy controls incubated with 1 µM NE compared to the participants of the HC group without NE incubation (*p* = 0.050) as well as between healthy controls incubated with 1 µM NE and ADHD cultures without NE incubation (*p* = 0.030). The *Per3* expression differed between controls and ADHD group at ZT28 (*p* = 0.012) (Fig. 2).

Although the *Bmal1* expression was strongly rhythmic in both groups, there was no ZT × group interaction via mixed ANOVA (Greenhouse–Geisser corrected *F*_0.36,26.40_ = 1.346, *p* = 0.122, partial ETA squared = 0.091). No ZT × group interaction via mixed ANOVA was observed for *Clock* (Greenhouse–Geisser corrected *F*_0.19,25.88_ = 0.866, *p* = 0.656, partial ETA squared = 0.064), *Cry1* (Greenhouse–Geisser corrected *F*_0.26,24.53_ = 1.084, *p* = 0.360, partial ETA squared = 0.075), *Per1* (Greenhouse–Geisser corrected *F*_0.73,27.34_ = 0.872, *p* = 0.654, partial ETA squared = 0.063) and *Per2* (Greenhouse–Geisser corrected *F*_0.87,18.18_ = 0.874, *p* = 0.269, partial ETA squared = 0.086) expression.

**Fig. 3:**
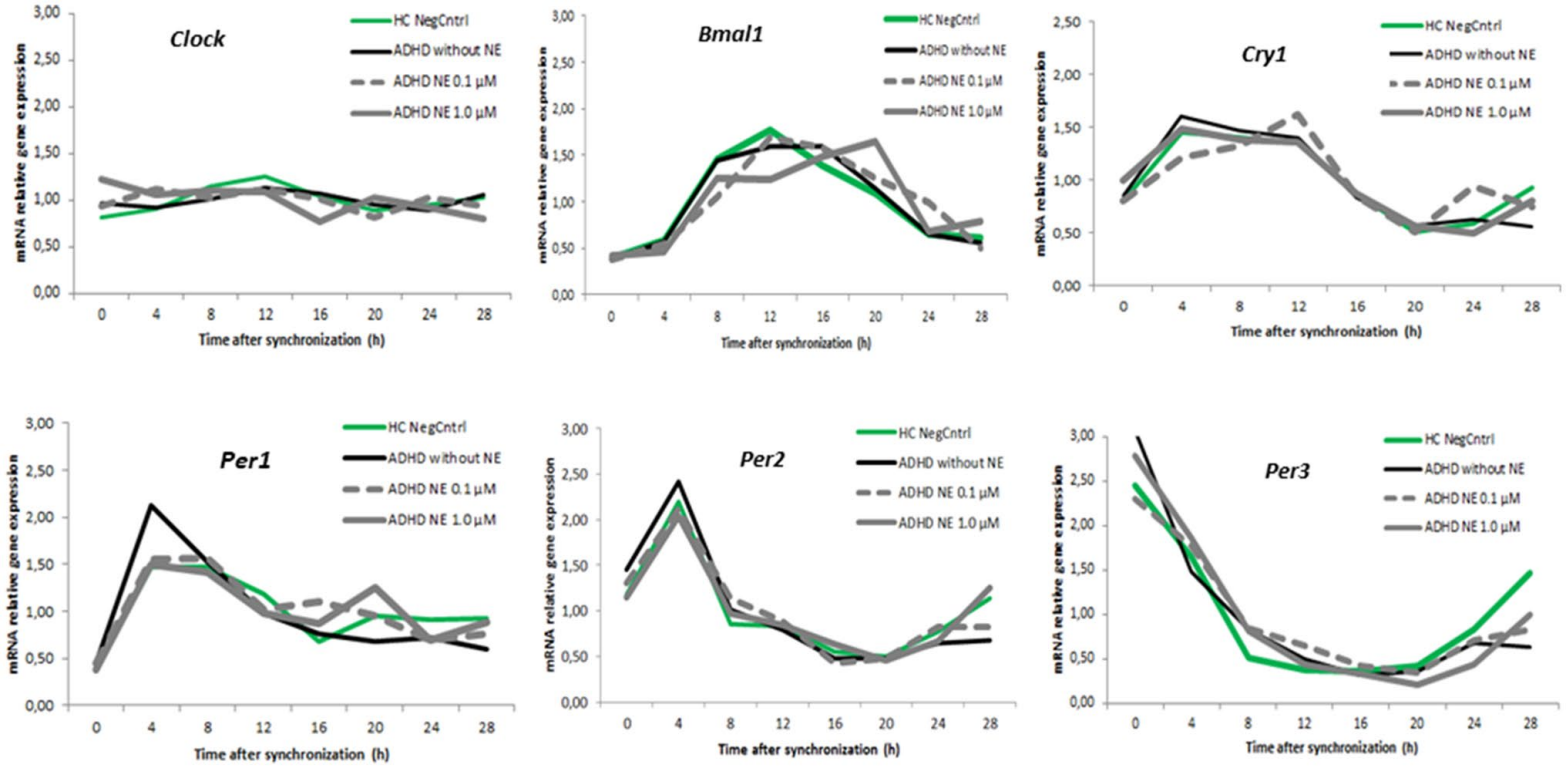
Relative mRNA gene expression of circadian genes in healthy controls (0 μM) and ADHD volunteers (0, 0.1, 1 μM NE)

*Clock*, *Bmal1* and *Cry1* resulted in a slight amplitude and phase differences between HC group and ADHD group after NE treatment, whereas, *Per1*, *Per2* and *Per3* after NE incubation lead to an adjustment to the expression of these *Per* genes to the control group (Fig. 3).

### Chronotype and circadian gene expression

58.3% of healthy participants displayed a neutral preference, whereas 25.0% presented moderate morning and 16.7% definite morning preference. Among the ADHD group 35.7% participants displayed moderate morning and 28.6% neutral preference. In the ADHD group, the evening preference was represented by 21.4% definite evening and 14.3% moderate evening type. The chronotype group data are presented in Table 3.

**Table 3:** Chronotype groups and demographic data in healthy and ADHD participants

Demographic data in healthy controls	All (*n* = 12)	Morning type		Neutraltype (*n* = 7)	Evening type		*p* value (one-way ANOVA)
		Moderate morning type (*n* = 3)	Definite morning type (*n* = 2)		Moderate evening type	Definite evening type	
Age (years)	41.50 ± 14.04	43.00 ± 15.72	56.00 ± 7.68	36.57 ± 12.83	N/A	N/A	ns
Gender	4 male, 8 female	3 female	2 female	4 male, 3 female	N/A	N/A	ns
BMI	25.86 ± 5.42	25.06 ± 4.05	25.55 ± 0.63	26.30 ± 6.91	N/A	N/A	ns
D-MEQ	58.83 ± 8.97	65.33 ± 2.88	72.00 ± 1.41	52.29 ± 3.95	N/A	N/A	9.10E − 05
WURS-k	7.17 ± 8.18	6.67 ± 7.02	1.00 ± 0.00	9.14 ± 9.44	N/A	N/A	ns
IQ	110.25 ± 9.32	106.67 ± 7.50	99.50 ± 6.36	114.86 ± 8.00	N/A	N/A	ns

Demographic data in ADHD	All (*n* = 14)	Morning		Neutraltype (*n* = 4)	Evening type		*p* value (one-way ANOVA)
		Moderate morning type (*n* = 5)	Definite morning type		Moderate evening type (*n* = 2)	Definite evening type (*n* = 3)	
Age (years)	41.57 ± 13.45	42.20 ± 15.55	N/A	45.50 ± 11.62	47.00 ± 16.97	31.67 ± 11.72	ns
Gender	9 male, 5 female	3 male, 2 female	N/A	2 male, 2 female	1 male, 1 female	3 male	ns
BMI	26.21 ± 3.64	26.15 ± 3.76	N/A	27.05 ± 4.78	26.40 ± 4.10	25.06 ± 3.42	ns
D-MEQ	46.57 ± 15.44	63.00 ± 3.08	N/A	47.50 ± 3.31	36.50 ± 2.12	24.67 ± 3.78	1.14E − 07
WURS-k	37.21 ± 15.20	41.00 ± 13.57	N/A	32.75 ± 10.63	17.00 ± 7.07	50.33 ± 13.86	ns
IQ	108.86 ± 12.60	106.80 ± 7.25	N/A	109.25 ± 19.61	120.00 ± 2.82	104.33 ± 13.43	ns

Differences of clock gene expression levels among chronotype were assessed using one-way ANOVA. Gene expression in healthy participants revealed a statistical significant difference between chronotypes for *Bmal1* at ZT16 (*F* = 4.884, *p* = 0.037) and *Per3* at ZT0 (*F* = 6.802, *p* = 0.016). A Bonferroni post hoc correction revealed a significant lower expression of *Bmal1* (*p* = 0.040) and *Per3* (*p* = 0.018) in healthy participants exhibiting neutral type compared to those with moderate morning type. In ADHD participants, *Bmal1* expression presented differences at ZT8 (*F* = 6.630, *p* = 0.013), particularly between moderate morning and definite evening type (Bonferroni post hoc test, *p* = 0.013). In HC cultures incubated with NE, one-way ANOVA revealed a statistical significant gene expression differences between chronotypes for *Per1* (with 0.1 µM NE, *F* = 6.680, *p* = 0.019) and *Per2* at ZT0 (with 0.1 µM NE, *F* = 6.802, *p* = 0.016), as well as *Clock* (with 1.0 µM NE, *F* = 4.639, *p* = 0.041) at ZT24. Bonferroni post hoc correction showed significant differences of *Per1* expression levels at ZT16 (*p* = 0.018) between neutral type and moderate morning chronotypes. When compared to definite morning chronotype, the expression of *Per2* at ZT16 (*p* = 0.031) and *Clock* at ZT24 (*p* = 0.050) was significant higher in healthy participants with neutral chronotype.

In ADHD cultures incubated with NE, several significant differences were observed. One-way ANOVA revealed a statistical significant differences in gene expression after 0.1 µM NE between chronotypes for *Clock* (*F* = 7.654, *p* = 0.006), *Cry1* (*F* = 7.365, *p* = 0.007), *Per1* (*F* = 5.747, *p* = 0.017) and *Per2* (*F* = 9.109, *p* = 0.003). Bonferroni post hoc analysis showed differences in expression between evening chronotypes. The participants with definite evening preference had a higher *Cry1* at ZT0 (*p* = 0.014) and *Per1* at ZT4 (*p* = 0.022), as well as a lower *Per1* at ZT24 (*p* = 0.039) compared to ADHD participants with moderate evening preference. In participants with moderate evening preference, the expression of *Per1* at ZT24 was significant higher compared to those exhibiting neutral (*p* = 0.005) and moderate morning preference (*p* = 0.012). Same differences were observed for *Per2* at ZT0 (*p* = 0.003) between the moderate evening and moderate morning chronotypes. Participants with an ADHD diagnosis with moderate morning chronotype revealed a higher *Clock* expression at ZT4 (*p* = 0.005) compared to moderate evening chronotype and a lower *Cry1* expression at ZT0 (*p* = 0.003) compared to definite evening chronotype. After 1 µM NE, *Per1* expression presented differences at ZT28 (*F* = 5.592, *p* = 0.023), particularly between moderate morning and moderate evening chronotypes (Bonferroni post hoc test, *p* = 0.024).

A Spearman’s rank order correlation showed the relationship between chronotype and clock gene relative expressions. There was a strong positive correlation for chronotype and *Bmal1* (ZT16, *r*_*s*_ = 0.611, *p* = 0.035), *Cry1* (ZT16, *r*_*s*_ = 0.591, *p* = 0.043) and *Per3* gene (ZT0, *r*_*s*_ = 0.631, *p* = 0.028) in HC cultures. In HC cultures incubated with NE, a strong positive correlation for chronotype with *Bmal1* (HC with 0.1 µM NE at ZT28, *r*_*s*_ = 0.611, *p* = 0.035; HC with 1 µM NE at ZT16, *r*_*s*_ = 0.670, *p* = 0.017) and *Per3* gene (HC with 0.1 µM NE at ZT0, *r*_*s*_ = 0.710, *p* = 0.010) was observed. In the ADHD group, chronotype and *Bmal1* gene expressions were positively correlated, at ZT4 (*r*_*s*_ = 0.714, *p* = 0.004) and ZT8 (*r*_*s*_ = 0.892, *p* < 0.0001), as well as *Cry1* (ZT8, *r*_*s*_ = 0.544, *p* = 0.044) and *Clock* at ZT12 (*r*_*s*_ = 0.620, *p* = 0.018). In the ADHD cultures incubated with NE, a positive correlation between chronotype and *Clock* (ADHD with 0.1 µM NE at ZT4, *r*_*s*_ = 0.574, *p* = 0.031), *Cry1* (ADHD with 0.1 µM NE at ZT4, *r*_*s*_ = 0.547, *p* = 0.043), *Per1* (ADHD with 1 µM NE at ZT20, *r*_*s*_ = 0.554, *p* = 0.040) and *Per2* (ADHD with 1 µM NE at ZT24, *r*_*s*_ = 0.628, *p* = 0.039) was observed.

## Discussion

The results of the present study illustrate that ADHD is associated to alterations in the circadian rhythm. It demonstrates that norepinephrine (NE) impacts significantly on circadian function. The expression and rhythm of all *Per* genes in the ADHD group adjusted to the healthy control (HC) group after NE incubation.

Previous studies have indicated that circadian processes are altered in neuropsychiatric disorders such as ADHD (Baird et al. 2012; Coogan et al. 2016, 2019; Coogan and McGowan 2017; Cronin et al. 2017; Faltraco et al. 2020; Johansson et al. 2016; Korman et al. 2018; Lippert et al. 2014; Mansour et al. 2017; McCarthy et al. 2013a; Yang et al. 2009). Our group reported that patients with ADHD using ADHD-medication (methylphenidate and atomoxetine) have altered sleep activity compared to both controls and ADHD participants without medication. At the molecular level, there were alterations in the expression of *Per2* and *Cry1* between ADHD individuals with no medication compared to medicated ADHD patients or HC, whereas *Clock* expression was altered in patients with ADHD using ADHD-medication (Coogan et al. 2019). The observed medication effect in the ADHD group is attributed to norepinephrine modulation. Second-line pharmacological treatment for ADHD besides stimulant methylphenidate, is atomoxetine (ATO), a selective *NET* inhibitor which increases NE levels. Extensive loss of NE terminals has been linked to alterations in brain regions which are vital for cognition, mood, and executive function (Phillips et al. 2016) as well as alterations of the circadian clock (Andrade-Silva et al. 2014; Li and Cassone 2015; Morioka et al. 2010; Terazono et al. 2003).

We observed several changes in the rhythmic expression of *Per3* gene. Cultures from ADHD patients with no NE resulted in lower expression of *Per3* 28 h after dexamethasone synchronization than either controls or cultures with NE. Expression of *Per3* was higher in the controls with 1 µM NE cultures at ZT4 compared to cultures without NE.

Animal studies demonstrated that alterations of *mBmal1*, *mPer1* and *mPer2* as well as *gBmal1*, *gClock*, *gCry1* and *gCry2* are associated with NE pathways (Li and Cassone 2015; Terazono et al. 2003), however, no alteration of *Per3* linked to NE has been reported until now.

Chronotype is an important influence on circadian clock gene expression (Pegoraro et al. 2015; Takahashi et al. 2018). In *Drosophila melanogaster*, gene expression was associated with early and late chronotypes (Pegoraro et al. 2015). Takahashi and colleagues observed that chronotype and social jetlag were related to the rhythm of clock gene expression in 24 young adults with a significant main effect of time for *Per3* and nuclear receptor subfamily *NR1D1* and *NR2D2* in the morningness group (Andrade-Silva et al. 2014; Li and Cassone 2015; Morioka et al. 2010; Takahashi et al. 2018; Terazono et al. 2003).

A variable number tandem repeat (VNTR) polymorphism in *Per3* (Dijk and Archer 2010) has been associated with chronotype, sleep homeostasis and various psychiatric disorders (Andrade-Silva et al. 2014; Li and Cassone 2015; Morioka et al. 2010; Terazono et al. 2003). No association between the *Per3* VNTR and diurnal preference was observed among Norwegian university students (Andrade-Silva et al. 2014; Li and Cassone 2015; Morioka et al. 2010; Terazono et al. 2003). Another study including one hundred and eighty eight healthy young adults, observed no effect for chronotype and circadian gene polymorphism of *Clock* and *Per3* (McGowan et al. 2020).

We observed changes in the circadian gene expression levels among healthy and ADHD participants with different chronotypes. After exposure of HDF cultures to NE, more significant differences were observed, particularly between ADHD participants exhibiting morning and evening preferences. Our results show a strong significant correlation of chronotype with gene expression 16 h after dexamethasone synchronization for *Bmal1* and *Cry1* genes, and immediately after synchronization for *Per3*. After incubation of HDF cultures obtained from healthy controls with NE, the positive correlation of *Bmal1* was shifted to 28 h after cell synchronization. In ADHD participants, the chronotype is positively correlated with *Clock*, *Bmal1* and *Cry1* genes in the first 12 h after synchronization. In the ADHD group, the exposure to NE reveals a positive correlation of chronotype with *Clock* and *Cry1* at ZT4, as well as with *Per1*/*2* at ZT20 and ZT24.

Some studies have suggested that chronotype is associated with weight gain that may induce changes in the circadian gene expressions. A downregulation of *Per1*, *Per2*, *Per3*, *Nr1d2* and PAR-domain basic leucine zipper transcription factors *Dbp*, *Tef* was observed in adipose tissues of mice after weight fluctuations (Dankel et al. 2014). A prospective study of weight gain associated with chronotype among college freshmen reports that individuals with eveningness chronotype have a significantly greater BMI compared with morningness and neutral types (Culnan et al. 2013). In our study, the healthy individuals with neutral chronotype have a slightly higher BMI (26.30 ± 6.91) compared to the morningness type (25.31 ± 2.34). This applies also to the ADHD participants, with neutral type having a higher BMI (27.05 ± 4.78) than both the morningness chronotype (26.15 ± 3.76) and the eveningness chronotype (25.73 ± 3.76).

A study by Lucassen et al. determined that adults who present an eveningness preference and sleep less than 6.5 h a day have more 24 h urinary epinephrine levels (Lucassen et al. 2013). In addition, the plasma and urinary NE are elevated in patients with obstructive sleep apnea syndrome (Fletcher 2003). A study reported circadian gene dysfunction in patients with obstructive sleep apnea syndrome, particularly changes in *Per1* mRNA expression (Burioka et al. 2008). In the same study, it was observed that administration of NE induced *Per1* mRNA in the cerebral cortex of mice *in vivo* (Burioka et al. 2008).

In summary, NE influences the circadian clock in human dermal fibroblasts from study participants with a diagnosis of ADHD.

It is to mention, that no special cognitive testing was implemented in this study. In addition, the participants of the ADHD group took no medication before and during the study. For further studies, a connection between circadian disturbances, cognitive deficits and the effect of medication would be suitable.

## Supplementary Information

Below is the link to the electronic supplementary material.

Supplementary file1 (DOCX 259 kb)

## Data Availability

Data and material are available.

## Code availability

Actiwatch 2, Philips Respironics, USA; CircWave v. 1.4 software (generated by Dr. Roelof Hut; http://www.euclock.org); SPSS (IBM Corporation).
