# Supplementary figures and images for: Norepinephrine influences the circadian clock in human dermal fibroblasts from study participants with a diagnosis of attention-deficit hyperactivity disorder
Source: J Neural Transm (Vienna). 2021 Jul 18;128(7):1147–57. doi: 10.1007/s00702-021-02376-2 (PMC8295072; doi:10.1007/s00702-021-02376-2)

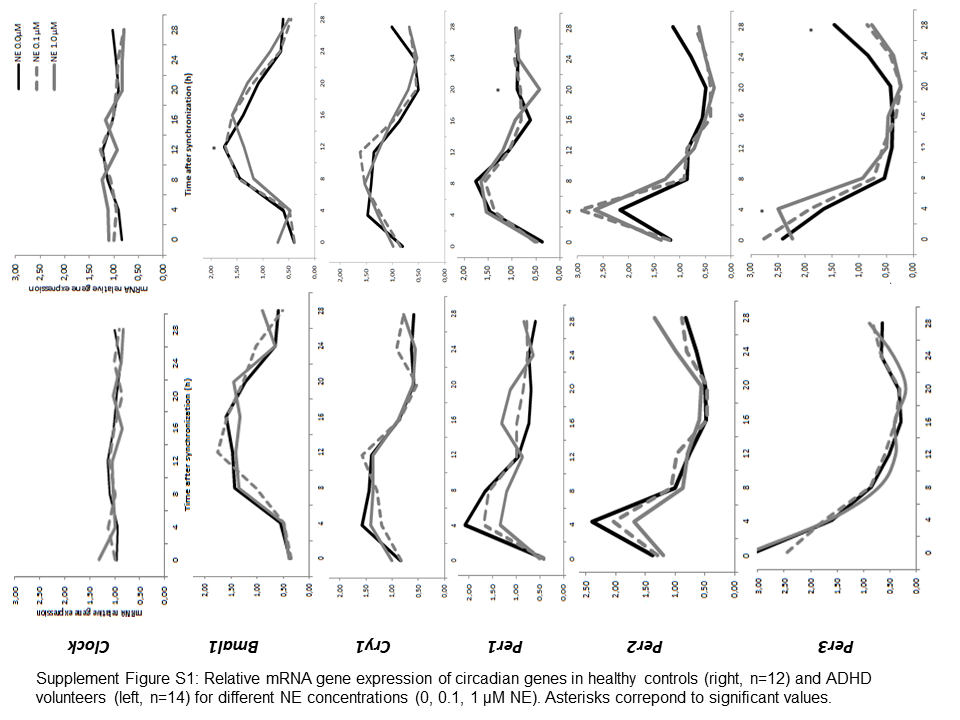

Supplement: Supplementary file 1 — Supplementary file1 (DOCX 259 kb) [file 702_2021_2376_MOESM1_ESM.docx]
